# The Evolution of the Dental Unit

**Published:** 1923-05

**Authors:** 


### THE EVOLUTION OF THE DENTAL UNIT

No man can practice dentistry with his bare hands. If he could, he wouldn't have to bother his head about equipment.

A dentist has his time to sell; but his time isn't worth anything without skill; and skill is of no value without material helps.

This is an age of conservation. We are attacking waste wherever we discover it. Sometimes it is hidden in the lost motion of inefficient equipment; in appliances that lack precision and that destroy the morale of the operator.

The Electro Dental Operating Unit was designed to eliminate all waste of effort and to concentrate under the operator's hand everything that a Unit can supply for precision and operating efficiency.

When consideration is given to all the elements contained in this Unit, and what they would cost separately—and then the conservation of time and effort is considered—its purchase should be regarded as a matter of purest economy.

At least, we think you owe it to yourself personally to investigate this Unit and see it demonstrated.

An interesting contemplation is the history of the so-called "Dental Unit." It happens (if it is a mere "happen") that the development of dental science and practice has coincided with the development of machinery. The best dentists have generally been good students of mechanical and electrical art; they have been interested in discovery, and have brought about a better adaptation of mechanism to the requirements of their practice than has been achieved by any others in the healing profession.

They have pointed the way in hydraulics and pneumatics; in the use of electricity for producing motion, for generating heat and light, for determining lesions and for modifying physiological function. We have seen the growth of the chair and the cuspidor; the air compressor, the blow pipe, the warm air syringe and the dental atomizer; the engine, the lathe and the mallet; the galvanic and faradic; the Violet Ray and the X-ray; the cataphoric and the ionizer; illuminators and transilluminators; and switchboards with their various applicators for diagnosis, therapeusis, esthesis and restoration.

Through a long period of growth, each of these mechanisms was developed to high efficiency and all were adopted, at one time or another, into the dental armamentarium; but they came into use at different periods, were necessarily constructed with little regard to each other and were set up wherever a support could be found. This resulted in the double irony of capable appliances incapable of satisfactory use—equipment of beautiful completeness, but of ugly appearance.

This was typical of the better grade dental office until recently, but such a condition could not, in the very nature of things, endure. This mechanical jumble demanded to be organized.

It had already been found that some of these appliances, though good in themselves, were not practical in dentistry, that others had too many joints and adjustments, and much eliminating and simplifying had been effected; so that, when the time came for a thorough organization of the dental operatory, the main problem was how to re-locate the essential apparatus so as to render all parts accessible to the dentist while working in the operative center of his surgery—the patient's mouth.

The solutions of this problem are seen in the several forms of the "dental unit." Most of these are partial, but are of value under certain conditions. One of the most complete solutions is that shown herein. In this all of the essential electrical and compressed air instruments, which it is practicable to group together, are arranged upon one base so as to meet the practical requirement of accessibility, the esthetic requirement of good looks and the sanitary requirement of cleanableness; all to a very satisfactory degree. It is particularly to be noted that certain operating instruments of great value are placed nearer the operative center than ever before.

The ideal of every operator is, of course, to have at hand the instruments best suited to each operation; and, since these are frequently to be found among those applicators actuated by electricity and compressed air, the ideal is brought appreciably nearer by this change in arrangement.

The bleaching of discolored teeth is now removed from the list of operations to be avoided, oral diagnosis is aided, prophylactic treatments are made more effective, therapeusis of pulpless teeth is facilitated, oral surgery is made safer, etc.

